# Nearby transposable elements impact plant stress gene regulatory networks: a meta-analysis in *A. thaliana* and *S. lycopersicum*

**DOI:** 10.1186/s12864-021-08215-8

**Published:** 2022-01-04

**Authors:** Jan Deneweth, Yves Van de Peer, Vanessa Vermeirssen

**Affiliations:** 1 Department of Plant Biotechnology and Bioinformatics, Ghent University, Ghent, Belgium; 2 VIB Center for Plant Systems Biology, Ghent, Belgium; 3 Department of Biochemistry, Genetics and Microbiology, University of Pretoria, Pretoria, South Africa; 4 Department of Biomedical Molecular Biology, Ghent University, Ghent, Belgium; 5 Department of Biomolecular Medicine, Ghent University, Ghent, Belgium; 6 Lab for Computational Biology, Integromics and Gene Regulation (CBIGR), Cancer Research Institute Ghent (CRIG), Ghent, Belgium

**Keywords:** Transposable elements, Gene regulation, Stress, Regulatory networks, Plant genomes

## Abstract

**Background:**

Transposable elements (TE) make up a large portion of many plant genomes and are playing innovative roles in genome evolution. Several TEs can contribute to gene regulation by influencing expression of nearby genes as stress-responsive regulatory motifs. To delineate TE-mediated plant stress regulatory networks, we took a 2-step computational approach consisting of identifying TEs in the proximity of stress-responsive genes, followed by searching for cis-regulatory motifs in these TE sequences and linking them to known regulatory factors. Through a systematic meta-analysis of RNA-seq expression profiles and genome annotations, we investigated the relation between the presence of TE superfamilies upstream, downstream or within introns of nearby genes and the differential expression of these genes in various stress conditions in the TE-poor *Arabidopsis thaliana* and the TE-rich *Solanum lycopersicum*.

**Results:**

We found that stress conditions frequently expressed genes having members of various TE superfamilies in their genomic proximity, such as SINE upon proteotoxic stress and Copia and Gypsy upon heat stress in *A. thaliana*, and EPRV and hAT upon infection, and Harbinger, LINE and Retrotransposon upon light stress in *S. lycopersicum*. These stress-specific gene-proximal TEs were mostly located within introns and more detected near upregulated than downregulated genes. Similar stress conditions were often related to the same TE superfamily. Additionally, we detected both novel and known motifs in the sequences of those TEs pointing to regulatory cooption of these TEs upon stress. Next, we constructed the regulatory network of TFs that act through binding these TEs to their target genes upon stress and discovered TE-mediated regulons targeted by TFs such as BRB/BPC, HD, HSF, GATA, NAC, DREB/CBF and MYB factors in Arabidopsis and AP2/ERF/B3, NAC, NF-Y, MYB, CXC and HD factors in tomato.

**Conclusions:**

Overall, we map TE-mediated plant stress regulatory networks using numerous stress expression profile studies for two contrasting plant species to study the regulatory role TEs play in the response to stress. As TE-mediated gene regulation allows plants to adapt more rapidly to new environmental conditions, this study contributes to the future development of climate-resilient plants.

**Supplementary Information:**

The online version contains supplementary material available at 10.1186/s12864-021-08215-8.

## Background

Transposable elements (TEs) form the major part of ‘junk DNA’ in all eukaryotic genomes. These DNA elements have the potential to be mobile and therefore to induce genomic changes and reshape genomes over the course of life [1]. Two major classes of TEs, several subclasses, superfamilies and families are distinguished based on their transposition mechanisms, sequence similarities and structural relationships [2–4]. In class I TEs, known as retrotransposons, transposition occurs through a ‘copy and paste’ mechanism with an RNA intermediate and the enzyme reverse transcriptase. Class I retrotransposons can be grouped into two subclasses. Long terminal repeats (LTRs) are characterized by the presence of long direct repeats at both ends and contain major superfamilies such as Gypsy and Copia that occur in most eukaryotes. Non-LTRs are in turn classified into long and short interspersed nuclear elements (LINEs and SINEs). Transposable elements of class II, also known as DNA transposons, replicate by a ‘cut and paste’ mechanism in the case of Terminal Inverted Repeats (TIR), or rolling circle in the case of Helitrons. Here, Mariner, Mutator/MuDR and hAT are examples of DNA transposon superfamilies that are widespread across the eukaryotic tree. Many of them contain Miniature Inverted-repeat Transposable Elements (MITEs), which are small non-autonomous TIRs that are present in high copies in genomes. Both classes of TEs can have autonomous and non-autonomous elements, where autonomous TEs encode all necessary products required for transposition [1, 5].

The genomes of most species, including plants, are dominated by TEs. There is a wide variety in the TE content between plant species and within species between individual plants [6, 7]. TEs account for about 21% of the reference genome in *Arabidopsis thaliana*, but this model organism is at the lower edge of the TE content spectrum, since most plant species have much higher numbers: 40% in rice, 60% in tomato, 80% in wheat and up to 85% in maize [5, 8, 9]. Especially retrotransposons constitute the predominating part of plant species with big genomes, such as tomato and maize.

Although TEs are major drivers of genome evolution and remnants of massive TE bursts are visible in plant genomes [9, 10], transpositional activity is largely prevented through epigenetic silencing by DNA methylation, histone modification and small RNAs in order to maintain genome integrity [11, 12]. Nevertheless, there is mounting evidence that TEs participate in the regulation of plant gene expression upon changing environmental conditions. Expression and transposition activity of quiescent TEs upon plant abiotic and biotic stresses is well known. Activation of TEs upon stress is often mediated through de-repression of the silenced epigenetic state or the activation by a transcription factor (TF) [13, 14]. Next, stress-activated TEs have the ability to alter the expression of genes flanking their insertion sites, which leads to phenotypic plasticity and adaptation to stress [15–17]. However, the relationship between stress and TEs is complex: some studies also report TE repression or harmful effects of TE activation upon stress [13]. Moreover, the genomic context of the site of TE insertion defines their specific role in gene regulation [11].

TEs can exert a regulatory role in host gene expression in multiple ways [15, 17]. Insertion of a TE in or near a gene can lead to new transcription start sites and promotor behavior [18], disrupt existing and/or create novel regulatory motifs [19, 20], or spread the chromatin state of the TE to the gene’s genomic context [21]. As a classroom example, the emergence of the melanism phenotype in British peppered moths during the industrial revolution is caused by a TE insertion into the first intron of the cortex gene that increases the abundance of the transcript [22]. In *A. thaliana*, the ONSEN LTR retrotransposon is activated in response to heat stress due to heat response factors recognizing a regulatory sequence in the promoter of ONSEN. As a consequence, the insertion of ONSEN was shown to induce the transcriptional upregulation of neighboring genes upon heat stress [19, 23]. Also in *A. thaliana*, the duplicated gene CYP82C2 underwent regulatory neofunctionalization through exaptation of a retroduplicated LINE retrotransposon into an enhancer, thereby creating transcriptional regulation by WRKY33 in the context of a pathogen defense metabolite biosynthetic pathway [20]. In tomato, the Rider Copia retrotransposon, which is triggered by drought stress and abscisic acid signaling, contains several environment-responsive cis-regulatory motifs, such as Dehydration Responsive Elements (DRE), in its promoter [24]. The above examples illustrate how single TEs can drive the evolution and diversification of stress gene regulatory networks (GRNs). TEs frequently contain transcription factor binding sites (TFBS) or regulatory motifs, which they spread through the genome by transposition [16]. With the public availability of plant genomes and high-throughput technologies, this phenomenon has also been investigated in a genome-wide manner. In this respect, as many as 85% of the sequences that fit the E2F binding motif are within MITEs in some Brassica species [25]. Furthermore, MITEs have amplified and mobilized the binding motifs of the bZIP60 and PIF3 TFs in peach and *Prunus mume*, and the TCP15/23 binding motif in tomato [26].

Two major plant studies have investigated genome-wide the influence of TEs on gene expression. Interestingly, a large-scale study in maize profiled gene and TE transcript levels in seedlings exposed to heat, salt, chilling and UV stress [27]. The analysis of TE families inserted within upstream regions of upregulated genes revealed that several, different TE families, including all major TE superfamilies such as TIRs, Gypsy, Copia and LINEs, are associated with upregulated gene expression in each of these stress conditions, affecting up to 20% of the upregulated genes, and as many as 33% of genes that are only expressed in response to stress. Expression of many of these same TE families also responds to the same stress conditions. In addition, the consensus sequence for binding of the abiotic stress responsive DREB/CBF TFs was found in most of these TEs in most stress conditions, suggesting that these TEs provide local enhancer activities that stimulate stress-responsive gene expression. Allelic variation for TE insertions is strongly associated with variation in stress-responsive gene expression, linking TEs to the adaptive stress response [27]. Similarly, in the context of fruit ripening in tomato, repeats are present in the majority of differentially methylated regions proximal to genes and several TEs including SINE and LINE elements, are enriched in the proximity of genes that are differentially regulated during ripening [8]. Hence, these studies suggest that TEs may contribute to the response of nearby genes to plant stress by providing stress-responsive enhancer-like functions and that the stress activation of TEs is highly context-dependent: the type of stress, the TE, its genomic location, the host genetic background all play a role.

Moreover, the contribution of TEs to the evolution of GRNs is not unique to plants, but is a conserved phenomenon across species, including mammals [28, 29].

Hence, TEs are frequently reactivated in response to stress and their activation can introduce TE copies into the genome with cis-regulatory motifs, enhancers or associated chromatin states that are responsive to stress, thereby rewiring GRNs. However, we still lack a comprehensive understanding of how TEs mediate GRNs in different plant species upon various stress conditions and different genomic positionings.

In this study, we systematically investigated the potential of TEs to function in the rewiring of stress GRNs in the TE-rich *Solanum lycopersicum* (tomato) and the relative TE-poor *Arabidopsis thaliana* using publicly available high throughput sequencing data of various stress conditions and extensive, structural annotations of their genomes. We focused on gene-proximal TEs that are implicated in differential gene regulation upon stress and their contribution to regulatory motifs that are bound by stress-responsive TFs. We considered the positioning of TEs relative to the gene, i.e. upstream, within introns or downstream, since the insertion site can influence the regulatory effect of the TE, and grouped TEs in superfamilies (TEFs). We constructed a compendium of RNA sequencing data of various stress conditions in Arabidopsis and tomato and processed them to produce lists of differentially expressed genes for each stress condition in every study. Next, we determined whether these genes, differentially expressed in a specific stress condition, were significantly enriched for TEFs located upstream, within intronic regions and downstream relative to non-differentially expressed genes and compared to expected frequencies of all TE-proximal genes (separate analyses for enrichment in up- or down-regulated genes for each stress). In both plant species, this lead to specific TEFs that are associated with specific stress conditions. Next, we searched for cis-regulatory motifs in the TEs adjacent to differentially expressed genes upon stress and constructed the stress GRNs that are mediated through these TEs.

## Results

### Positioning of TEs relative to genes in *A. thaliana* and *S. lycopersicum*

Inspired by the work of Makarevitch for maize abiotic stress and Jouffroy for tomato ripening [8, 27], we aimed to investigate genome-wide the association of TE families with stress-responsive expression of nearby genes in various stress conditions in Arabidopsis and tomato. We grouped TEs in superfamilies (TEFs) to reduce the complexity of the analysis on one hand and to be able to reveal overarching patterns on the other hand. We only considered TEs in well-defined superfamilies and ignored the superfamilies ‘Unknown’, ‘Unassigned’, ‘Confused_TE’ and containing putative TEs (Methods).

**Fig. 1:**
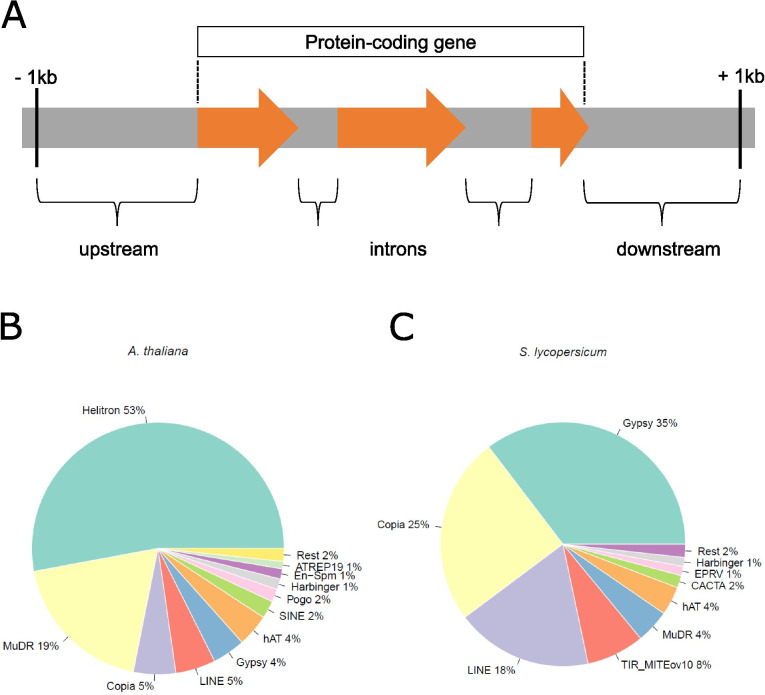
Positioning and abundance of TEs nearby protein-coding genes. **A**) Three genomic positionings of TEs relative to genes were considered: “upstream” contains TEs within 1 kb upstream of the gene, “downstream” contains those within 1 kb downstream of the gene, and “intron” contains TEs within introns. Only the transposon boundary closest to the gene was considered for this classification. **B**) Well-defined TE superfamilies (TEFs) adjacent to protein-coding genes in *A. thaliana* and the proportion of gene-proximal TEs they hold. ‘Rest’ indicates the sum of Mariner, ATDNA12T3_2, Tc1 and ATREP18 TEFs. **C)** Well-defined TE superfamilies (TEFs) adjacent to protein-coding genes *in S. lycopersicum* and the proportion of gene-proximal TEs they hold. ‘Rest’ indicates the sum of Mariner, Retrotransposon, Helitron and TRIM_LARD TEFs

Gene-proximal TEs might influence gene expression differently depending on their positioning in respect to the gene [8]. Therefore, we considered three different genomic positionings for TEF adjacency to genes (Fig. 1A): within 1 kb upstream, inside introns and within 1 kb downstream of a gene, thereby avoiding overlap with other genes. We chose a maximal distance of 1 kb, since in the TE richer and bigger maize genome, at least half of all genes have an overlapping TE or a TE within 1 kb upstream [30].

The Araport11/TAIR10 annotation of the *A. thaliana* genome contains 27,420 protein-coding genes and 31,189 TEs, while the ITAG3/SL3.0 annotation of the *S. lycopersicum* genome includes 33,697 protein-coding genes and 531,409 TEs (Table 1). Considering all protein-coding genes in the genome and all three genomic positionings of gene-proximal TEs, we found a total of 14,420 TEs near Arabidopsis genes and 59,236 TEs near tomato genes that are classified in well-defined superfamilies (Methods). The most abundant TEFs adjacent to genes are Helitron and MuDR for *A. thaliana*, summing up to about 70% of all gene-proximal TEs (Fig. 1B). In *S. lycopersicum*, Gypsy, Copia and LINE make up about 80% of all gene-proximal TEs (Fig. 1C) (Table S1).

**Table 1:** Number of genes and TEs in the genomes of *A. thaliana* and *S. lycopersicum.* For well-defined TE superfamilies (TEFs) the number of TE-proximal genes and the number of TEs in the different genomic positionings with respect to their nearby protein-coding genes are given

	Genes	TE-proximal genes	UP	IN	DOWN	Gene-proximal TEs	Total TEs
*A. thaliana*	27,420	9540	10,099	2163	6997	14,420	31,189
*S. lycopersicum*	33,697	22,949	17,175	39,507	15,668	59,236	531,409

Genes is the total number of protein-coding genes annotated in the genome of the species. ‘TE-proximal genes’ is the total number of protein-coding genes adjacent to well-defined TEFs. Upstream (UP), intragenic (IN) and downstream (DOWN) refer to the relative positioning of TEs to adjacent genes, up to a maximum distance of 1 kb. ‘Gene-proximal TEs’ is the unique total number of TEs in well-defined TEFs adjacent to protein-coding genes, hence a combination of the upstream, intragenic and downstream positioning, where the same TE can be in different genomic locations for different genes. ‘Total TEs’ gives the total number of TEs annotated in the genome of the species

Hence, while in Arabidopsis the Class II DNA transposons are highly represented in TEs adjacent to protein-coding genes, in tomato we mostly found Class I retrotransposons near protein-coding genes. In addition, roughly half of all TEs in well-defined TEFs are adjacent to protein-coding genes in the gene-dense genome of *A. thaliana*, as compared to about 10% of all TEs in *S. lycopersicum*. About one third of Arabidopsis protein-coding genes and almost 70% of tomato protein-coding genes have TEs in their proximity, and due to the higher TE and gene-proximal TE content for tomato, tomato has still more than double TEs adjacent to its TE-proximal genes. Whereas for *A. thaliana* most gene-proximal TEs reside in the upstream genomic positioning, for *S. lycopersicum* most gene-proximal TEs were found in introns, which can likely be explained by the presence of more and larger intronic sequences in *S. lycopersicum* than in *A. thaliana*.

### Genes differentially expressed upon stress are enriched for specific TEFs nearby

From the SRA database, we downloaded high-quality RNA-seq data from 20 experimental conditions and 9 studies in *A. thaliana* and 33 experimental conditions and 17 studies in *S. lycopersicum* (Table S2). After preprocessing, we calculated consensus differential expression using the well-recognized packages DESeq2 and EdgeR, based on the comparison between experimental and control replicates for each specific stress condition (see Methods, Table S2). After filtering for conditions with at least 100 consensus differentially expressed genes, we ended up with 15 experimental conditions in *A. thaliana* and 25 in *S. lycopersicum* for further analysis (Table S2), encompassing different abiotic and biotic stresses such as drought, salt, cold, heat, paraquat, photorespiratory, proteasome and infection stresses for Arabidopsis and cold, heat, light, hormone, stress mutant and various infection stresses for tomato. In *A. thaliana*, the amount of differentially expressed genes ranged from roughly 1000 to over 8000 genes. For tomato, this ranged from 500 to several thousands of differentially expressed genes. One study in tomato, encompassing several light conditions, consistently reported about 14,000 differentially expressed genes, a large chunk of all expressed genes. There were generally about as many genes upregulated as downregulated for any particular study.

**Fig. 2:**
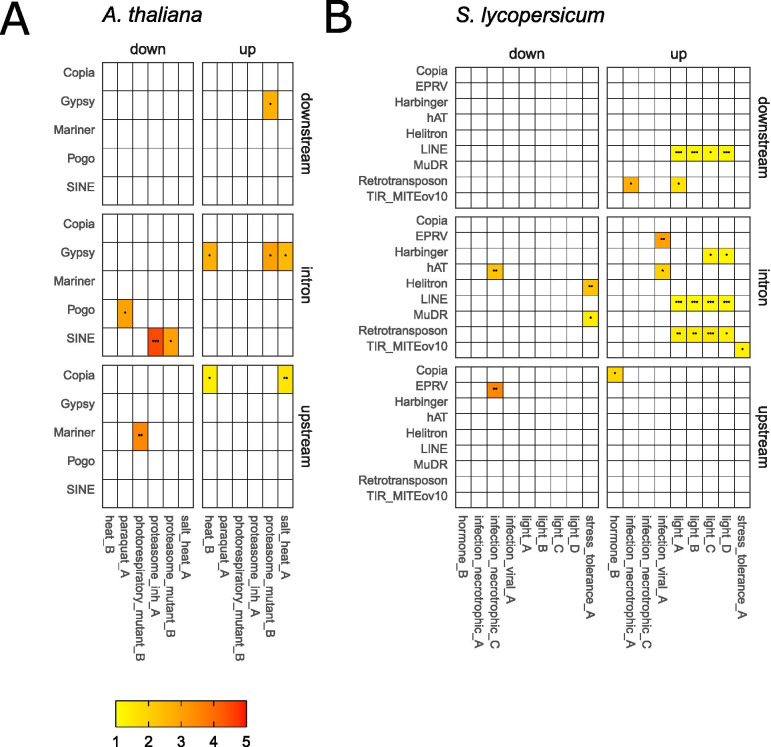
Fold enrichment of differentially expressed genes near specific TE superfamilies (TEFs) upon stress for **A**) *A. thaliana* and **B**) *S. lycopersicum*. The Chi-squared test was conducted separately for up- and downregulated genes with TEFs upstream, downstream or within introns. The intensity from yellow to red reflects the enrichment score with values between 1 and 4.5, as compared to all differentially expressed genes near all TEFs in that specific genomic positioning and stress condition. The significance of enrichment is indicated within the tiles: * = FDR adjusted *p*-value < 0.05, ** = FDR adjusted p-value < 0.01, *** = FDR adjusted p-value < 0.001. We additionally filtered out significant results for which the observed number of differentially expressed genes near a TEF was less than 5 and the expected number was less than 2. Only TEFs, stress conditions and genomic positionings for which a valid enrichment was found are shown. ***A. thaliana***: heat_B = 1 h incubation at 44 °C - leaves, paraquat_A = spray with 25 μM paraquat, photorespiratory_mutant_B = SHORT_ROOT (*shr*) mutant – 24 h photorespiratory stress, proteasome_inh_A = 100 μM proteasome inhibitor MG132, proteasome_mutant_B = *rpn-10* mutant – RPN10 is a subunit of the 26S proteasome, salt_heat_A = 150 mM NaCl for 15 days + 1 h incubation at 44 °C – leaves. ***S. lycopersicum***: hormone_B = 48 h after treatment with ACC (ethylene precursor), infection_necrotrophic_A = infection by *Colletotrichum gloeosporioides* - leaves, infection_necrotrophic_C = infection by *Pseudomonas syringae* pv. tomato DC3000 - leaves, infection_viral_A = infection by Tomato yellow leaf curl virus - leaves, light_A = constant shade – shoot apical meristem / leaf primordia, light_B = constant sun – shoot apical meristem / leaf primordia, light_C = sun to shade – shoot apical meristem / leaf primordia, light_D = constant sun - shoot apical meristem / leaf primordia, stress_tolerance_A = male-sterile, stress tolerant mutant

As TEs can provide regulatory motifs and rewire GRNs, we characterized genome-wide relationships between TEFs and their adjacent genes, as well as associations between TEFs and stress conditions of the gene expression meta-analysis. We used a similar methodology as previously described [8, 27], but at TE superfamily instead of TE family level, and we conducted a more stringent and comprehensive analysis by looking only at protein-coding genes, applying more rigorous statistical criteria and considering more stress conditions and 2 species (see Methods). For the set of genes located in a specific genomic positioning near a specific TEF, we calculated the likelihood that this set is enriched for either differentially up- or downregulated genes using the Chi-squared Goodness of Fit test, using the probabilities of all differentially and non-differentially expressed TE-proximal genes in the same genomic positioning as the expected distribution. We applied this procedure for each genomic positioning of TEF-gene relations (upstream, intron and downstream), each TEF and each stress condition. Upon a significant test, the output is an enrichment of specific TEFs located upstream, within introns or downstream of differentially expressed genes in a specific stress condition (Fig. 2, Fig. S2). This enrichment could point to the specific retention and function of these TEs as regulatory motifs in the differential expression upon a specific stress.

For Arabidopsis, we detected 10 enrichments of specific TEF adjacent differentially expressed genes for both up- and downregulated genes and in all three genomic positionings (Table S3). In this plant genome fewer gene-proximal TEs are present for TE-proximal protein-coding genes (Table 1). About 7-60% of genes with specific TEFs nearby were stress-responsive, as compared to about 3-20% for all genes near TEFs, reaching enrichment scores up to 4.5 (Table S3). We detected significant enrichments upon heat (heat_B) and a combination of heat and salt stress (salt_heat_A) for upregulated genes with Copia TEs in their upstream regions and Gypsy TEs in their introns (Fig. 2A). We found the Copia and Gypsy superfamily enriched for upregulated genes in two independent heat stress experiments, further contributing to the relevance of our observations. Looking at the TE family level for Copia, these upstream regions mostly contained META1, ATCOPIA30 and ATCOPIA78 elements. The Gypsy intronic elements contained mainly TEs from the families ATHILA7, ATHILA6A and TAT1_ATH. Furthermore, we identified enrichments for Gypsy TEF, including ATLANTYS3, ATGP3, ATGP9B and ATHILA4A, both in downstream regions and introns of upregulated genes upon proteotoxic stress (proteasome_mutant_B). For the downregulated genes, we found significant enrichments for Pogo TEF, mostly ATHPOGON1, in intronic regions upon paraquat stress (paraquat_A); SINE TEF, including RathE1_cons, RathE3_cons and RathE2_cons, in intronic regions upon proteotoxic stress (proteasome_inh_A and proteasome_mutant_B) and Mariner, especially DT1, in upstream regions upon photorespiratory stress (photorespiratory_mutant_B).

For tomato, we detected 24 significant enrichments of specific TEFs adjacent to differentially expressed genes upon stress in multiple stress conditions and in all genomic positionings (Fig. 2B). About 1-57% of genes with specific TEFs nearby were stress-responsive, as compared to about 1-41% for all genes near TEFs, reaching enrichment scores up to 3.5 (Table S3). Also here, TEFs within introns were the most prominent and upregulated genes had more adjacent TEF than downregulated genes. In the different light conditions, we observed the presence of the same TEFs near upregulated genes in intronic regions, some of which were also observed in downstream regions: Harbinger, LINE and Retrotransposon. Strong enrichment scores were observed for EPRV, Retrotransposon and hAT in the intronic, but also upstream and downstream regions of up- and downregulated genes in multiple infection conditions. More specifically, we found enrichments for hAT within introns of and for EPRV upstream of downregulated genes upon infection by *Pseudomonas syringae* pv. tomato DC3000 (infection_necrotrophic_C) and for hAT and EPRV within introns of upregulated genes upon infection by the tomato yellow leaf curl virus (infection_viral_A). Upon infection by *Colletotrichum gloeosporioides* (infection_necrotrophic_A), the Retrotransposon superfamily, containing retrotransposons other than Gypsy, Copia and LINE, was enriched downstream of upregulated genes. In addition, we obtained enrichments in a male-sterile, stress tolerant mutant, for both Helitron and MuDR TEFs in the introns of downregulated genes and for TIR_MITEov10 in the introns of upregulated genes. Also, upon ethylene treatment, upregulated genes contained Copia TEF in their upstream regions.

We also calculated GO Biological Process enrichment for the differentially expressed genes for the specific stress conditions for which an association with TEF positioning was observed. The detected functional enrichments were in line with the specific stress conditions under study (Table S4).

Overall, we detected significant TEF-gene associations, both in Arabidopsis and tomato, in specific genomic positionings and in specific stress conditions, highlighting the potential of these repeat elements to act as regulatory motifs in differential gene expression upon stress. Several enrichments were supported by multiple experiments for a specific stress and are therefore more likely to be relevant.

### Regulatory motif detection in stress-responsive TEs

To connect the stress-responsive genes adjacent to TEFs to a potential regulatory function of TEs, we searched for TFBS in sequences of enriched TEF members near DE genes. We investigated regulatory motifs in the genomic positioning for which an enrichment was found through de novo and known plant cis-regulatory motif detection using the RSAT tools peak-motifs and dna-pattern respectively (Methods) [31]. Overrepresentation of the motif in the TE sequence is assessed against a background set of sequences and a differential E-value (peak-motifs) or *p*-value (dna-pattern) is calculated. In order to use a background set of sequences that is similar to the test set, all other TE sequences in the same genomic positioning were taken as background. Hence, using this approach we aimed to find specific cis-regulatory motifs related to specific TEF-stress associations.

In our case, de novo motif detection by peak-motifs was based on oligo-analysis, which identifies the overrepresentation of words based on word size seeds of 6 and 7 as compared to all TE sequences near genes in the specific genomic positioning [32]. We compared the identified motifs to known TFBS from the Cistrome, footprintDB-plants, JASPAR core non-redundant plants and cisBP motif databases. These cis-regulatory motif databases are somewhat biased to the model plant *A. thaliana*, which has to be taken into account for the analysis on tomato. An overview of the most significant motifs detected de novo by peak-motifs is depicted in Tables 2 and 3. The significance of the motifs cannot be directly compared between different genomic positionings, because of different statistical backgrounds, and between different stress conditions, because of different numbers of up- or downregulated genes and associated TEFs. Interestingly, a significant motif could be assigned for most stress condition associated gene-proximal TEFs. Homeobox or homeobox-like motifs (ZHD, HD, SANT/MYB) were highly significantly picked up within Pogo TEF in introns of downregulated genes upon paraquat stress in Arabidopsis. In a male-sterile, stress tolerant mutant of tomato, the best predicted motif within TIR_MITEov10 TEs in the introns of upregulated genes was predicted to be bound by REM19, an AP2/ERF/B3 TF. Moreover, similar motifs were detected for similar stress conditions. The GAGA-motif for BARLEY B RECOMBINANT / BASIC PENTACYSTEINE (BBR/BPC) TFs as well as C2H2 zinc fingers (RAMOSA1), and the binding motif for GATA TFs (ZML2, ZML1, GATA15) were detected within Copia TEF for heat stress and combined salt and heat stress in Arabidopsis, upstream of stress-responsive genes. For tomato, we identified the same cis-regulatory motifs in Harbinger TEs within the introns of stress-responsive genes in multiple light conditions: an unknown motif and a MYB motif. We observed similar motifs for LINE TEFs in introns (unknown, NAC, HAP3, MYB) and downstream (unknown, MYB) of upregulated genes in multiple light conditions. Also within Retrotransposon TEs in the introns and downstream of light upregulated genes in multiple conditions, the same motif for the AP2/ERF/B3 TF RAV1 was found. Hence, not surprisingly, we sometimes identified a similar motif for the same TEF for different genomic positionings upon a specific stress. Upon proteotoxic stress in *A. thaliana*, binding motifs for Heat Shock Factors (HSF) and/or S1Fa-like TFs were identified within Gypsy TEF both in the intronic regions as well as downstream of upregulated genes.

**Table 2:**
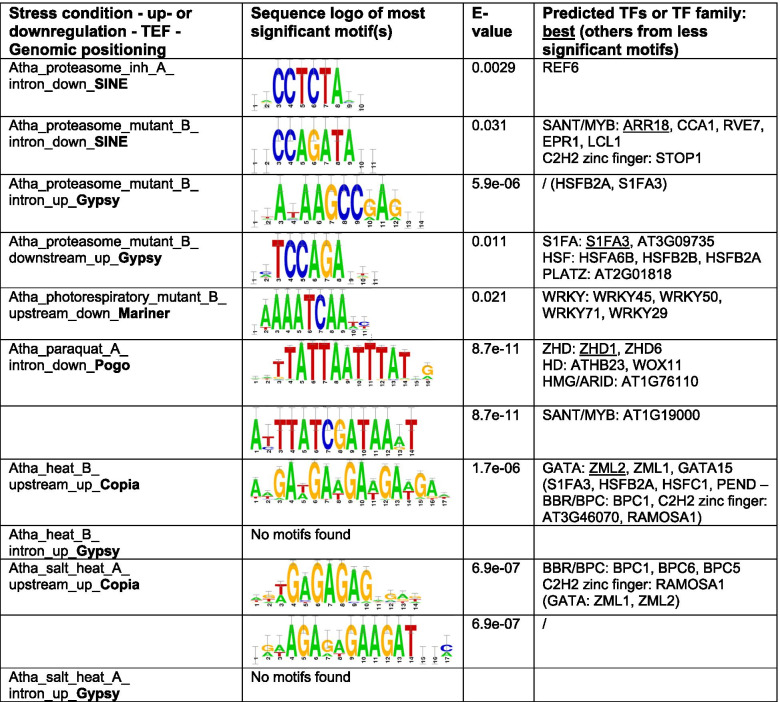
Most significant cis-regulatory motifs detected de novo by RSAT peak-motifs in TE sequences adjacent to stress-responsive genes in *A. thaliana*

We only analyzed sequences of enriched TEF members near stress-responsive genes in a specific genomic positioning (upstream, intron, downstream). All other TE sequences in the same genomic positioning were taken as background. Detected motifs were compared to the motif databases Cistrome, footprintDB-plants, JASPAR core non-redundant plants and cisBP. Only most significant sequence logo(s) are displayed. N.S. = non-significant

**Table 3:**
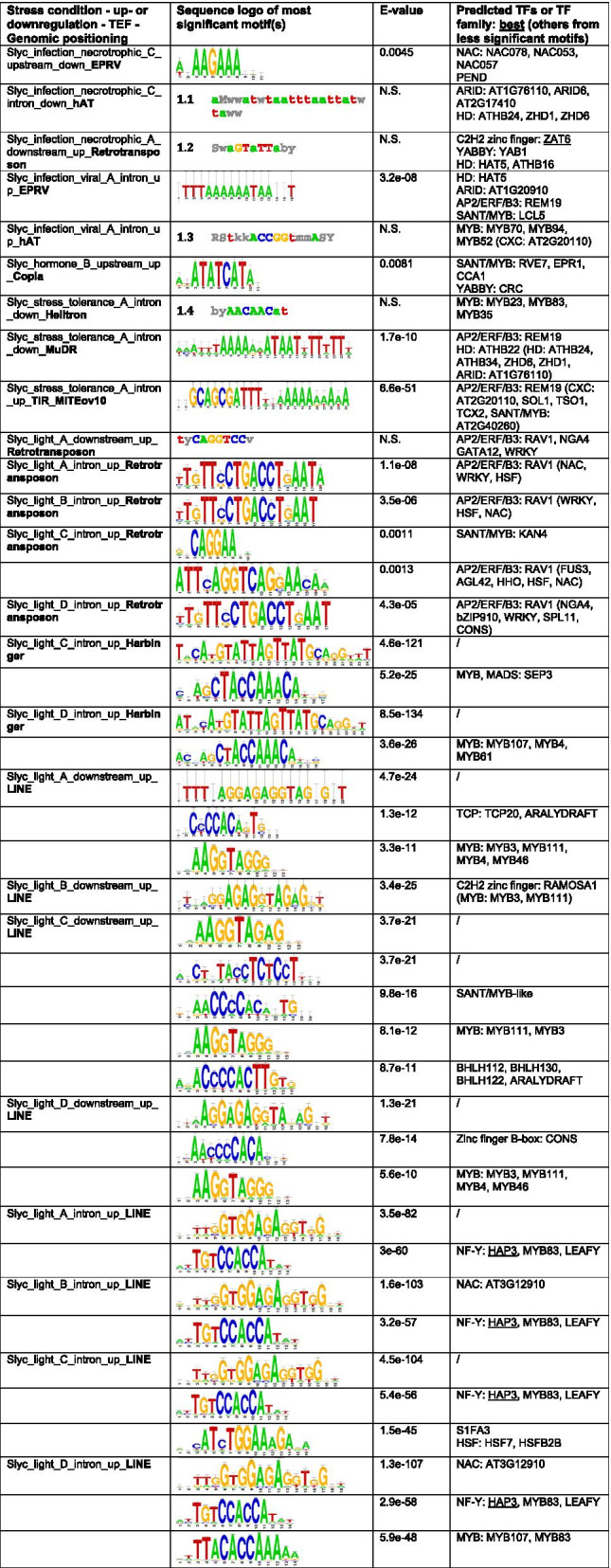
Most significant cis-regulatory motifs detected de novo by RSAT peak-motifs in TE sequences adjacent to stress-responsive genes in *S. lycopersicum*

We only analyzed sequences of enriched TEF members near stress-responsive genes in a specific genomic positioning (upstream, intron, downstream). All other TE sequences in the same genomic positioning were taken as background. Detected motifs were compared to the motif databases Cistrome, footprintDB-plants, JASPAR core non-redundant plants and cisBP. Only most significant sequence logo(s) are displayed. N.S. = non-significant

For several of the TFs or regulatory factors matching the de novo motifs, we found support for stress-responsive signaling for the specific stress conditions under study. Regarding the heat and salt stress conditions in *A. thaliana*, BPC1/BPC2 are reported to positively affect salt tolerance in *A. thaliana* [33], ZML2 and ZML1 TFs have been reported to function in the cry1-mediated photoprotective response [34] and GATA factors control cold tolerance [35]. Regarding HSFB2A and Arabidopsis proteasome mutant, HSF-driven induction of ubiquitin genes upon proteotoxic stress has been reported in human cell lines [36]. Regarding infection in tomato, the homeodomains ATHB22 and ATHB25 function in the tolerance of seed deterioration to virus infection in Arabidopsis [37]. RELATED TO ABI3 AND VP1 (RAV) proteins act as transcriptional repressors and are involved in light responses [38].

We also detected novel motifs with no hits in the plant motif databases, such as in Gypsy for proteotoxic stress and Copia for salt-heat stress in Arabidopsis and in Harbinger and LINE for light stress in tomato.

Next, we searched the same TE sequences for 2735 known plant TFBS from footprintDB, AGRIS, PLACE and the literature and calculated stringently their overrepresentation as compared to all TEs in the same genomic positioning with at least one motif (Methods) (Table S5). We provide an overview of the most enriched and most relevant TFBS in TEFs adjacent to stress-responsive genes in Table 4.

**Table 4:** Most significant plant cis-regulatory motifs detected by RSAT dna-pattern in TE sequences adjacent to stress-responsive genes for *A. thaliana* (Atha) and *S. lycopersicum* (Slyc)

Sample	Motif pattern	Name	Percentage in gene-proximal TEs (%)	Enrichment	Adjusted p-value
Atha_proteasome_mutant_B_ intron_down_**SINE**	GTTAGGTTC	ACIII element (MYB)	17	49.4	0.0174
Atha_proteasome_mutant_B_ intron_up_**Gypsy**	tACACGbmACyk	NAC019	20	177.8	0
	vyaCACGgmAcyr	NAC055	20	177.8	0
	aYACGCAA	NAC080	50	22.2	5.83E-06
	mrCACGTGyk	MYC4 (BHLH)	20	118.5	3.45E-05
	rrCACGTGyy	ILR3 (BHLH)	20	59.3	0.00049
Atha_proteasome_mutant_B_ downstream_up_**Gypsy**	GTGGaCCCrs	TCP16	10	889	0
	TACCGACGA	DRE-like	10	889	0
	GGCCGACGT	DRE-like	10	592.7	0
	mrCACGTGyk	MYC4 (BHLH)	10	592.7	0
	dwwkvhsACGTGKCa	GBF3 (bZIP)	10	444.5	0
	vGAAssTTCy	**HSFB2A**	10	63.5	0
Atha_photorespiratory_mutant_B_upstream_down_**Mariner**	CAATGATTG	AtHB5	29	36.8	0.0005
	CAATSATTG	AtHB2	29	36.8	0.0005
	yCAATCAWtg	AtHB7	29	29.6	0.0009
	wAATATATTw	AHL20 (AT-hook)	57	4.5	0.0136
Atha_paraquat_A_ intron_down_**Pogo**	wawawAAATATCtwa	AT3G10113 (**SANT/MYB**)	14	84.7	0.0265
	aAAATATCTt	CCA1 (**SANT/MYB**)	29	16.9	0.0322
	awycTTATCtthwy	AT3G11280 (**SANT/MYB**)	14	50.8	0.0322
	AGAAATTTCT	HSEs binding site motif	14	28.2	0.0449
	TACGTACAA	SBP-box (zinc finger)	14	31.8	0.0449
Atha_heat_B_ upstream_up_**Copia**	ACAGAG	REF6	32	2.3	0.0088
	TGGGCY	SITEIIATCYTC (TCP)	25	2.1	0.0326
	ayACGywAy	AtNAC6	13	2.8	0.0394
Atha_heat_B_ intron_up_**Gypsy**	AGCCGACGA	DRE-like	11	65.9	0.0180
Atha_salt_heat_A_ upstream_up_**Copia**	ACAGAG	REF6	28	2.1	0.0212
	ayACGywAy	AtNAC6	14	2.9	0.0224
	GGGCC	SORLIP2	22	2.3	0.0228
	TGGGCY	SITEIIATCYTC (TCP)	25	2.1	0.0237
Atha_salt_heat_A_ intron_up_**Gypsy**	AGCCGACGA	DRE-like	9	53.9	0.0295
Slyc_infection_necrotrophic_C_ upstream_down_**EPRV**	GATAAGR	I-box core	71	3.7	0.0664
Slyc_infection_necrotrophic_C_ intron_down_**hAT**	wAAwwwwTTw	AHL12 (AT-hook)	94	3.9	1.03E-09
	rTTTAAAh	TCX6 (CXC)	72	3.6	1.60E-05
	rTTTrAAw	SOL1 (CXC)	83	2.7	2.98E-05
	dAwTTAAwTw	AGF1 (AT-hook)	56	5.0	3.38E-05
	rwWAAmGT	COG1 (DOF)	78	2.7	0.0001
Slyc_infection_necrotrophic_A_ downstream_up_**Retrotransposon**	CCAATAAAGG	CArG-box (MADS)	13	69.7	0.0343
	CCTTTATTGG	CArG-box (MADS)	13	69.7	0.0343
Slyc_infection_viral_A_ intron_up_**EPRV**	yaahawhwwCAmCAACawyahh	AT1G18960 (**SANT/MYB**)	10	135.5	0.0037
	wwwwwTdACCGTTrr	MYB3R1 (**SANT/MYB**)	10	125.4	0.0037
	wthwwwACCGTTA	LOF2 (**SANT/MYB**)	10	80.6	0.0068
	GGCCGACAA	DRE-like	10	69.1	0.0068
	tmayTAATyAhgwww	ZFHD2	10	51.3	0.0101
Slyc_infection_viral_A_ intron_up_**hAT**	ATATTTAWW	SEF1MOTIF	67	5.4	4.57E-06
	wAAwwwwTTw	AHL12 (AT-hook)	83	3.4	5.42E-06
	tAWWTAWWta	AHL13 (AT-hook)	56	4.6	0.0001
	AAATTAAA	Bellringer/replumless/pennywise (AG/HD)	56	4.5	0.0001
	ATtwawaATTwAATt	AT1G76110 (HMG/ARID)	11	78.4	0.0002
	dACCGGTw	**MYB94**	11	7.1	0.0379
Slyc_stress_tolerance_A_ intron_down_**MuDR**	wwwCGhATwWT	AtHB32 (**HD**)	12	3.6	0.0338
	kATGTTGC	TEM2 (**AP2/ERF/B3**)	17	2.7	0.0414
	AAATTAAA	Bellringer/replumless/pennywise (AG/**HD**)	29	2.3	0.0220
	TTWTWTTWTT	MARTBOX	39	2.3	0.0042
	TTNCGTA	NAC binding site	24	2.2	0.0402
Slyc_light_A_ downstream_up_**Retrotransposon**	CATTAATTAG	Soybean homeodomein leucine zippers (GmHdl56, GmHdl57)	18	56.1	8.62E-06
	TTTTACTAGT	SORLREP1	14	29.9	0.0008
	yGCCGCC	ERF2 (tobacco)	23	8.7	0.0033
	rCACGTGy	BHLH3	18	10.8	0.0039
	ywTTTACyGc	BRADI1G77610 (MYB)	14	13.3	0.0066
Slyc_light_A_ intron_up_**Retrotransposon**	CATTAATTAG	Soybean homeodomein leucine zippers (GmHdl56, GmHdl57)	13	50	7.02E-15
	dwwGAAATGAwr	AT2G31460 (auxin response factor 70)	16	5.6	1.68E-05
	KWGTGRWAAWRW	GT-1 motif rbcS (pea)	11	2.7	0.0440
	wgawAAmGt	DOF4.7	17	2.1	0.0491
	wtcaGTTr	AtMYB87	21	2.0	0.0225
Slyc_lightB_ intron_up_**Retrotransposon**	CATTAATTAG	Soybean homeodomein leucine zippers (GmHdl56, GmHdl57)	12	44.7	1.23E-11
	dwwGAAATGAwr	AT2G31460 (auxin response factor 70)	12	4.3	0.0048
Slyc_light_C_ intron_up_**Retrotransposon**	CATTAATTAG	Soybean homeodomein leucine zippers (GmHdl56, GmHdl57)	15	55.3	1.84E-15
	dwwGAAATGAwr	AT2G31460 (auxin response factor 70)	15	5.3	0.0002
	waATgAtTAh	YAB5 (YABBY)	11	4.0	0.0087
Slyc_light_D_ intron_up_**Retrotransposon**	CATTAATTAG	Soybean homeodomein leucine zippers (GmHdl56, GmHdl57)	15	54.6	2.18E-15
	dwwGAAATGAwr	AT2G31460 (auxin response factor 70)	15	5.2	0.0002
	waATgAtTAh	YAB5 (YABBY)	11	4.0	0.0101
Slyc_light_C_ intron_up_**Harbinger**	AACCAAAC	**MYB binding site**	15	3.2	0.0002
	ACCAAAC	**MYB4**	27	2.6	7.97E-06
	rymAGTTA	**AtMYB4**	32	2.1	4.21E-05
	TATTAG	CPBCSPOR	51	2.0	4.27E-08
Slyc_light_D_ intron_up_**Harbinger**	TATTAG	CPBCSPOR	53	2.1	2.02E-10
	rymAGTTA	**AtMYB4**	31	2.1	2.85E-05
	ACCAAAC	**MYB4**	27	2.6	3.29E-06
	CATGCAT	RY repeat motif (soybean)	18	2.0	0.0134
	AACCAAAC	**MYB binding site**	15	3.3	4.67E-05
	CTAACCA	**AtMYB2**	14	2.1	0.0283
	rwakATtCyc	**GAMMAMYB2**	11	3.4	0.0007

We analyzed the overrepresentation of 2735 known plant TFBS collected from footprintDB, AGRIS, PLACE and the literature (Methods) in sequences of enriched TEF members near stress-responsive genes in a specific genomic positioning (upstream, intron, downstream) as compared to all TE sequences near genes in that genomic positioning. To limit false positives, we only considered motifs that were present in at least 10% of the TEs and that were at least two times overrepresented. We here display only the 5 most significant known motifs, in addition to any de novo detected motifs or relevant stress-related motifs. Matching TF families between the tools peak-motifs and dna-pattern are highlighted in bold

Using stringent selection criteria to reduce false positives (Methods), we identified significantly overrepresented TFBS for 9 out of 10 *A. thaliana* TEF-stress associations and for 13 out of 24 *S. lycopersicum* TEF-stress associations. Supporting the de novo motif detection from peak-motifs, we found TFBS for the same TF or TF family for several *A. thaliana* (HSF, SANT/MYB) and *S. lycopersicum* (SANT/MYB, HB, AP2/ERF/B3, MYB) TEF-stress associations. For example, we identified a HSFB2A motif within Gypsy TEs downstream of upregulated genes upon proteotoxic stress in Arabidopsis and a MYB94 motif within hAT in introns of upregulated genes upon viral infection in tomato by both peak-motifs and dna-pattern. In addition, we found additional motifs with dna-pattern. Again, we noted a high resemblance in motifs between similar stress conditions and TEFs in both species e.g. REF6, ATNAC6 and SITEIIATCYTC in Copia TEs upstream of both salt_heat_A and heat_B stress-responsive genes; DRE motifs in Gypsy TEs within introns of salt_heat_A and heat_B stress-responsive genes; soybean homeodomain leucine zippers, AT2G31460, YAB5 in Retrotransposon TEs within introns of upregulated genes upon light stress; MYB TFs in Harbinger TEs within introns of upregulated genes upon light stress. We identified several known stress-responsive motifs that corresponded to the specific stress condition such as the DRE motif upon heat stress in Arabidopsis, which can be bound by DREB/CBF TFs that function in heat stress responses [39], and SORLREP1 and GT-1 motifs upon light stress in tomato, which are known to be involved in light-regulated gene expression [40, 41]. Also, the TFs or members of the TF family that bind the detected motifs in stress-responsive TE sequences seem to function in the specific stress condition. We identified the NAC motif within Gypsy in introns of upregulated genes upon proteotoxic stress in Arabidopsis, where NAC TFs are known to play a regulatory role in maintaining protein homeostasis upon proteotoxic stress [42]. The observation of the REF6 motifs within Copia upstream of upregulated genes in heat_B and salt_heat_A conditions in Arabidopsis is supported by the fact that upon heat HSFA2 directly activates the H3K27me3 demethylase RELATIVE OF EARLY FLOWERING 6 (REF6), which in turn derepresses HSFA2 in a feedback loop [43]. Regarding the light conditions in tomato, homeodomain leucine zipper (HD-Zip) TFs are plant specific TFs with a role in responding to environmental stresses [44]. Arabidopsis MYB4 is well known as a key regulator in UV tolerance for its negative role in UV sunscreen biosynthesis, which explains why the MYB4 motif was discovered in Harbinger TEs within introns of upregulated genes in multiple light conditions in tomato [45].

Furthermore, several of the hits in the motif databases point to factors involved in DNA methylation and chromatin remodeling, which might be connected to the epigenetic regulation of TEs and genes upon stress. REF6 is a Jumonji-type histone demethylase that is thought to mediate the temporal and spatial de-repression of genes and its four Cys2His2 zinc fingers directly recognize a CTCTGYTY motif within active chromatin states [46, 47]. In addition, CXC domain proteins including TCX5 and TCX6, which transcriptionally repress genes required for DNA methylation maintenance, and SOL1/SOL2 and TSO1, which function in cell cycle progression, are part of the Arabidopsis DREAM complex that precludes DNA hypermethylation and organizes cell fate transitions [48, 49]. Finally, ARID domain factors belong to PEAT complexes that mediate histone deacetylation and heterochromatin condensation and thereby facilitate heterochromatin silencing [50].

Through a systematic cis-regulatory motif detection in TE sequences of TEFs adjacent to stress-responsive genes in different genomic positionings, we obtained many significant TFBS for stress-responsive TFs. Hence, these constitute novel hypotheses of how regulatory factors are coopted to stress-regulated regulons by TE activation upon stress.

### Gene regulatory networks mediated by TE cis-regulatory motifs

**Fig. 3:**
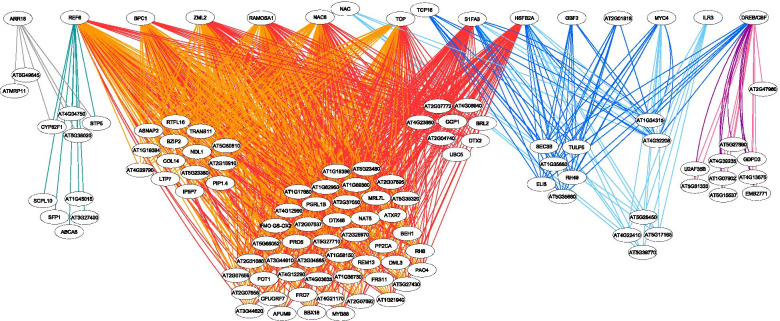
TE-mediated heat and proteotoxic stress gene regulatory network for *A. thaliana*. Copia elements in upstream regions and Gypsy elements in introns of heat-responsive genes recruited specific regulatory factors. Also, Gypsy elements within introns and downstream regions and SINE within introns of proteoxic stress-responsive genes hosted cis-regulatory motifs targeted by specific TFs. We can distinguish several regulons, related to the different TEF-differentially expressed genes associations from left to right: SINE/proteasome mutant targeted by ARR18 (grey), SINE/proteasome inhibitor targeted by REF6 (green), Copia/salt-heat targeted by BPC1, ZML2, REF6, NAC6, TCP and RAMOSA1 (orange), Copia/heat targeted by BPC1, ZML2, REF6, NAC6, TCP and RAMOSA1, in addition to HSFB2A and S1FA3 (red), Gypsy downstream/proteasome mutant targeted by HSFB2A, S1FA3, TCP16, AT2G01818, DREB/CBF, GBF3 and MYC4 (darkblue), Gypsy intron/proteasome mutant targeted by HSFB2A, S1FA3, NAC, MYC4 and ILR3 (lightblue), Gypsy/heat targeted by DREB/CBF (purple) and Gypsy/salt-heat targeted by DREB/CBF (pink)

For Arabidopsis, we constructed the GRNs mediated by the TEFs and stress conditions for which we had multiple experimental conditions available (Copia and Gypsy TEs upon heat stress, SINE and Gypsy upon proteotoxic stress) considering common and highly overrepresented binding sites for 15 TFs and/or TF families identified through our TFBS detection analysis (Fig. 3). From the different regulons that we observed, it is clear that TE-proximal gene sets are shared between similar conditions and different genomic positionings for the same TEF i.e. heat_B and salt_heat_A conditions both for Copia and Gypsy, between proteasome_mutant_B and proteasome_inh_A for SINE and between Gypsy within introns and downstream regions in proteasome_mutant_B. In addition, for all of the experimental conditions, specific stress-TEF regulons were found. Moreover, we detected TFs and/or TF families that target both differentially expressed genes in heat and proteotoxic stress such as REF6, HSFB2A and S1FA3, as well as DREB/CBF, NAC and TCP. These TFs and TF families are known to regulate multiple stress processes in plants [51–53]. Previously, we constructed an Arabidopsis stress GRN through reverse engineering of microarray expression data [54]. There, HSFB2A e.g. is also a predicted regulator of several modules implicated in different abiotic stress responses, including heat and proteotoxic stress.

## Discussion

In this study, we have systematically investigated the involvement of TEs as providers of regulatory motifs in stress GRNs of the plant species *A. thaliana* and *S. lycopersicum*. Over time, TEs have supplied multiple cis-regulatory motifs to plant regulons: MITEs have distributed binding sites for E2F, bZIP60, PIF3 and TCP15/23 [25, 26], Helitrons for PHE1 [55] and Copia for SEP3 [56]. Especially upon environmental stress, where TE upregulation occurs, TEs can influence the expression of nearby genes through contributing cis-regulatory motifs or associated chromatin states and as a result provide adaptation to stress [15]. As an example, the rice mPing MITE causes up-regulation of nearby genes in response to cold or salt stress [57]. Makarevitch conducted a pioneering study in maize to analyze the effect of adjacent TEs on the gene expression response to environmental changes and revealed that up to nine different TEFs are associated with upregulated gene expression upon heat, salt, cold and UV stress [27]. We used a similar approach in our meta-analysis, but included more stress conditions and compared two plant species, and different genomic positionings, as the regulatory effect of TEs can differ depending on the location of their insertion site relative to the nearby gene [8, 11]. For an overarching and less complex analysis at the systems level, we grouped TEs in superfamilies (TEFs). To delineate the TEF-mediated stress GRNs in Arabidopsis and tomato, we used a 2-step computational approach. First, we investigated if genes that have a member of a specific TEF either upstream, downstream or within its introns, are enriched for differential regulation in a specific stress condition, as compared to all genes near TEs in that specific genomic positioning and stress condition. This stringent analysis resulted in a set of conditions, associated with specific TEFs, and their up- or downregulated genes and adjacent TEs, similar to previous efforts [8, 27]. Recently, a web-based tool, called PlanTEenrichment was developed to calculate the enrichment analysis of TEs located within the upstream regions of a gene list within 11 plant species. However, this analysis is restricted to the upstream regions of genes and to 1000 genes at a time and considers individual TEs [58]. We assume that these stress-responsive TEFs contribute cis-regulatory motifs to their adjacent genes to rewire their expression and enable phenotypic plasticity upon stress. Hence, we searched for cis-regulatory motifs in the sequences of TEs belonging to these stress-responsive TEF-condition associations using a de novo motif prediction algorithm and a pattern search algorithm using well-recognized plant TFBS and rigourous statistical criteria to limit false positives. By linking the detected cis-regulatory motifs to known regulatory factors, we were able to construct the GRNs between the stress-responsive genes and these regulatory factors. Similar approaches to construct GRNs through non-TE cis-regulatory motifs or ChIP-seq peaks in upstream regions of genes are well-known and have resulted in biologically relevant GRNs [59, 60]. Despite the fact that we conduct both steps at the superfamily level of TEs and for publicly available expression data from various stress conditions and studies, this overarching analysis resulted in significant enrichments of specific TEF adjacent DE genes and overrepresented cis-regulatory motifs within TE sequences in specific stress conditions and genomic positionings. While some studies also directly scan TE sequences for known TFBS [25, 26], other recent studies in Drosophila and human have investigated the contribution of TEs to cis-regulatory motifs through the computational analysis of ChIP-seq peaks, whether or not in combination with RNA-seq analysis [29, 61]. In the ideal situation, one starts off from the stress experiment and measures both gene and TE expression, as well as binding of regulatory factors, followed by functional validation using genome-editing tools such as CRISPR/Cas9 to test the functional relevance of TEs and their binding motifs on putative target genes in the stress condition [62]. As we use a combination of stress-responsiveness by differential expression analysis and DNA binding by cis-regulatory motif detection, we conduct a thorough computational investigation to generate hypotheses on TE-mediated stress GRNs in Arabidopsis and tomato.

The comparison of the TE-poorer Arabidopsis plant to the TE-richer tomato plant resulted in some interesting similarities and differences. For both *A. thaliana* and *S. lycopersicum*, we detected statistical enrichment of differentially expressed genes near TEFs primarily in the intronic genomic positioning, more for upregulated genes and retrotransposon TEFs. Through a stringent statistical enrichment test, significant enrichments were detected for TEF-differentially expressed genes associations in several stress conditions. Moreover, we often observed the same TEF in similar stress conditions. The number of differentially expressed genes in a specific stress condition and the number of TE adjacent genes in a specific genomic positioning have an effect on the result of the statistical enrichment test. We tried to limit the first by only withholding conditions with at least 100 consensus DE genes. Due to the background of all TE adjacent genes in a specific genomic positioning, our test is stringent and likely to pick up TEFs that are very specific in a specific stress condition as compared to other gene-proximal TEFs. However, it is less likely to detect highly abundant TEFs in the species of interest. For example, we did not observe any TEF enrichment within differentially expressed upon stress for Helitron in Arabidopsis or Gypsy for tomato, which are the most abundant TEFs in the respective species. Looking at the family level instead of the superfamily level is an option here. Using the Araport/TAIR10 and the ITAG3/SL3.0 genome annotation for respectively Arabidopsis and tomato, we also only detect ‘relatively young’ TEs. More sophisticated computational tools are needed to extend the analysis to older, conserved TEs with more degenerated sequences, which have been shown to increase the TE content in the Arabidopsis genome up to 50% [56]. Moreover, a limitation of our study is that we have relied on the reference genomes for the TE annotation. For *A. thaliana*, all stress experiments relate to the reference Columbia-0 accession. For *S. lycopersicum*, however, different cultivars have been used in the stress studies. Nevertheless, according to a recent study, the majority of the annotated TEs in the tomato reference correspond to ancestral TE copies, while more recently mobilized TEs, are only present in one or a few tomato accessions and constitute TE insertion polymorphisms (TIPs) [6]. Likely, we will have missed these TIPs in this study.

We observed 10 enrichments of specific TEF adjacent differentially expressed genes for Arabidopsis. One study encompassing two heat stress conditions [63], revealed enrichment of the TEFs Copia and Gypsy, respectively in the upstream and intronic genomic positioning of upregulated genes. Within the upstream regions of heat stress upregulated genes, we mainly detected overrepresented cis-regulatory motifs for BRB/BPC, GATA, HSF, REF6, NAC and TCP factors within Copia TE sequences and DREB/CBF TFs in Gypsy TE sequences. For most of these regulatory factors, there is evidence from literature that they are involved in the plant heat stress response (see Results). In *A. thaliana*, the Copia ONSEN LTR retrotransposon has been shown to contribute heat-responsive elements (HREs) that are bound by Heat Shock Factors (HSF) to adjacent genes upon heat stress [19, 23]. In seven Brassicaceae species, the heat-responsiveness of COPIA families, mainly ONSEN, COPIA37, TERESTRA, and ROMANIAT5, is correlated with the presence of putative high affinity HSF binding HREs within their long terminal repeats [64]. Furthermore, heat stress induced TE activation correlates with global 3D chromatin organization rearrangement in Arabidopsis [14]. The latter study found retrotransposons such as Copia to be significantly overrepresented in heat-activated TEs, with the ONSEN/ATCOPIA78 being the most enriched. Correspondingly, we also identified several ATCOPIA78 elements in our heat-stress upregulated – adjacent to Copia TEs gene sets. Upon proteotoxic stress in Arabidopsis, enrichment for SINE within the introns of downregulated genes was identified in two experiments, one with a proteasome mutant and one with a proteasome inhibitor. Upon cis-regulatory motif finding in these adjacent TEs, binding motifs for REF6 and SANT/MYB factors such as ARR18 (Arabidopsis RESPONSE REGULATOR 18) were detected. ARR18 has been implicated in Arabidopsis in cytokine signaling and as a positive osmotic stress response regulator together with bZIP TFs [65].

For tomato, we detected 24 significant enrichments of specific TEFs adjacent to differentially expressed genes upon stress in stress conditions related to infection, stress tolerance, hormone and light, and within TEs located mainly in introns.. Again here, we observed the same TEFs associated with similar stress conditions within and across studies: EPRV and hAT within introns and upstream of up- and downregulated genes of several infection conditions, LINE elements within introns and in the downstream positioning of upregulated genes in light stress conditions, Harbinger and Retrotransposons within introns of light-responsive upregulated genes. In the context of tomato ripening, LINE elements have previously been associated with stress-responsive up- and downregulated genes as well [8]. In different tomato accessions, TE insertions polymorphisms from most superfamilies, including LINE and hAT, are found preferentially within or near genes and are associated with extreme variation in major agronomic traits or secondary metabolites [6]. Upon cis-regulatory motif detection, we detected significant overrepresentation of HD, ARID, CXC, MYB, SANT/MYB, NAC and AT-hook binding motifs within TE sequences of EPRV and hAT in multiple infection-related conditions. In multiple light conditions, AP2/ERF/B3 (RAV1), MYB, HD leucine zippers, AT2G31460 and YABBY motifs were enriched in Retrotransposon TEF members located near upregulated genes. Harbinger and LINE TEF members near light upregulated genes were also found to contain MYB motifs. Within LINE near light-responsive genes, also binding motifs for NF-Y and NAC TFs were discovered. In addition, novel, highly overrepresented motifs were identified in these TEFs in multiple light conditions. As the motif databases focus primarily on Arabidopsis, some caution should be taken when drawing conclusions from results in tomato, although DNA binding is somewhat conserved at the TF family level. Hence, for most of the associated regulatory factors in tomato, there is also evidence from literature that they are involved in specific stress responses (see Results).

## Conclusions

Over the years, several studies have reported evidence for the evolutionary, regulatory role of TEs in plant GRNs, especially upon environmental stress [8, 19, 20, 23–27, 64]. We provide here a significant contribution to the field by conducting a systematic meta-analysis on the contribution of TEs to stress-responsive cis-regulatory motifs and hence stress GRN rewiring in Arabidopsis and tomato using a 2-step computational approach. We observed both known and novel TF-TE motif-stress regulon associations and discovered biologically relevant connections at the TE superfamily and TE family level. In conclusion, TE-mediated gene regulation provides a powerful mechanism for plants to adapt more rapidly to new environmental conditions and the study of TE-mediated stress gene regulatory networks offers important insights into this process.

## Methods

### Transposable element annotation in superfamilies

TEs were annotated according to Araport11/TAIR10 for *A. thaliana* (www.arabidopsis.org/download/ > Genes > Araport11 genome release > Archives > Araport11_GFF3_genes_transposons.201606.gff.gz) [66, 67] and ITAG3/SL3.0 for *S. lycopersicum* (ftp.solgenomics.net/tomato_genome/annotation/ITAG3.0_release/ ITAG3.0_REPET_repeats_agressive.gff), downloaded May 2020 [8, 68]. We excluded TEs that were labelled ‘transposable_element_gene’ or ‘transposon_fragment’ for *A. thaliana* and that contained ‘Host Gene’ for *S. lycopersicum*. TEs were categorized in superfamilies (referred to as TE families or TEFs) for Arabidopsis as documented at TAIR using TAIR10_Transposable_Elements.txt. We excluded the ‘Unassigned’ TEs, as well as the ‘Unknown’ superfamily, as they contain different, unrelated TEs. Instead, we kept the most abundant families within the ‘Unknown’ superfamily i.e. ATREP18, ATDNA12T3_2 and ATREP19, which had over 150 copies in the *Arabidopsis thaliana* genome. The tomato annotation at ITAG3/SL3.0 already included the superfamily for each TE. We excluded the ‘Unclassified’, ‘Confused_TE’, ‘PutativenonAutoClassII’, ‘putNA_hAT’, ‘putNA_CACTA’, and ‘putNA_MuDR’ superfamilies, as the identities and/or classification of these TEs is not clear. We also removed the SAT and SSR superfamilies, since these repeats are not classified as TEs [4] and absent in the TE superfamily categorization of *Arabidopsis thaliana*. In this way, we created a repeat GFF3 file for each species, which was next converted to bed format using BEDOPS (v.2.4.32).

### Defining different genomic regions of protein-coding genes

The intronic, 1 kb upstream and 1 kb downstream regions of a gene were defined using BEDtools (v.2.27.1) substract, flank and intersect, after converting the genome annotation GFF3 files to bed format using BEDOPS (v.2.4.32) and taking into account not to overlap any other genes. Finally, we filtered these bed files to output protein-coding genes only.

### Preprocessing of RNA-seq data

We utilized publicly available, stress related RNA sequencing datasets for both *A. thaliana* and *S. lycopersicum* at the Sequence Read Archive (SRA). To be included in the final selection, studies had to be sufficiently clear in their method of treatment, include controls and at least two replicates, and be run on ILLUMINA sequencers. We used 20 conditions from 9 studies for *A. thaliana*, and 33 conditions from 17 studies for *S. lycopersicum*. The full overview of the SRA data used in this study can be found in Table S2 and an overview of the computational analysis is given in Fig. S1^1^. To remove likely adapter sequences and perform general trimming of reads to improve their quality, we applied Trimmomatic (v. 0.32) with the following parameters: SE or PE, −phred33, ILLUMINACLIP:: <adapter_sequences_file containing common ILLUMINA adapter sequences>:2:30:10 LEADING:10 TRAILING:10 SLIDINGWINDOW:4:20 MINLEN:35 [69]. We compared the quality before and after preprocessing using FastQC (v. 0.11.2) and removed low quality reads [70]. Next, reads were mapped to the genome using the seed-extent spliced aligner GSNAP (v. 2015-06-23), with the following arguments: -novelsplicing = 1, −localsplicedist = 15,000, −max-mismatches = 5 [71]. An index of the genome assembly of each species was built using the command “gmap_build -d <species> <genome_assembly>”, with the optional argument k, determining k-mer size of the index, left to its default of 15. We checked the quality of the read mapping using the tool Qualimap (v. 2.1) [72]. HTSeq (v. 0.6.1) with specific arguments -t exon -i Parent -s no, took the BAM alignment files generated by GSNAP and a GFF3 formatted genome annotation (Araport11/TAIR10 anotation for Arabidopsis, ITAG SL3.0 annotation for tomato) to produce gene counts [73]. Reads that could be mapped to multiple identifiers (ambiguous) or that have been mapped to multiple places in the genome (not unique) were effectively ignored. Finally, we removed genes that were not “sufficiently” expressed i.e. had a count below the cut-off equal to the sum of 10 and the number of stress and control replicates for a given stress condition.

### Differential gene expression

We applied two statistical R packages to predict which genes are differentially expressed in stress versus control conditions EdgeR (v. 3.16.5) and DESeq2 (v. 1.14.1 )[74, 75]. Both are based on methods using the negative binomial distribution. For DESeq2, we simply used the builtin ‘DESeq’ function with default parameters. For EdgeR, all steps are performed explicitly: input was normalized using ‘calcNormFactors’, dispersion was estimated by sequentially applying the functions ‘estimateGLMCommonDisp’, ‘estimateGLMTrendedDisp’, ‘estimateGLMTagwiseDisp’, models were fit using ‘glmFit’ and the LRT test was applied using ‘glmLRT’ on the fitted models. The results for EdgeR are retrieved using the ‘topTags’ function. In order to produce more accurate results, we combined both DESeq2 and EdgeR by selecting the consensus or intersection of the results with adjusted *p*-values less than 0.05 with the Benjamini-Hochberg multiple hypothesis testing correction from both tools. We filtered the consensus to contain protein-coding genes only. Conditions that had less than 100 consensus DE genes were removed from further analysis, remaining studies had over 500 consensus DE genes each.

### Enrichment analysis for TE family proximal differentially expressed protein-coding genes

We built upon methodology used by Makarevitch for maize abiotic stress and Jouffroy for tomato ripening [8, 27]. To associate specific TEs and TE superfamilies (TEFs) to protein-coding genes, we used intersectBed from BEDtools (v.2.27.1) on the intronic, 1 kb upstream and 1 kb downstream gene regions bed files and the repeat bed files. Only the TE boundary closest to the gene was considered for the specified genomic region. To evaluate whether a specific set of genes, adjacent to a TEF, was enriched for differentially expressed (DE) genes, we used the Chi-squared Goodness of Fit test (customized Python (v. 3.7.3) script with the ‘stats.chisquare’ function of the SciPy module (v. 0.15.0)). Significantly up- and downregulated genes were tested separately. The test was given as input the observed frequencies of DE, i.e. either up- or downregulated, genes, adjacent to a specific TEF and non-DE genes near a specific TEF and the expected frequencies of DE/non-DE given all expressed genes near all TEFs. Since we tested multiple TEFs per condition for enrichment of DE genes, *p*-values for each condition were adjusted with the Benjamini-Hochberg multiple hypothesis testing correction and the significance level was set at 0.05. We discarded test results when the observed number of differentially expressed genes near TEF was lower than 5 or the expected number was lower than 2, as the Chi-squared test gives inaccurate results when the numbers become too small. We also performed an exact test of goodness-of-fit (‘stats.binom_test’ function of the SciPy module (v. 0.15.0)) and taking the above filtering on observed and expected numbers into account, obtained largely the same results as with the Chi-squared test (data not shown). We calculated the fold enrichment score of observed over expected frequencies for TEF adjacent DE genes. Further processing was done with the R packages tidyverse and biomaRt.

### Gene ontology analysis

Biological Process GO enrichment was analyzed through the R packages GOstats (v.2.40.0) [76]. The gene-GO annotation table for the species of interest was limited to Biological Process terms and downloaded for Arabidopsis from TAIR and for tomato from the PANTHER database on 15/05/2017. We constructed a gene set collection for all GO annotations of a species using the GOstats methods ‘GOFrame’ and ‘GOAllFrame’ consecutively on the gene-GO annotation table, followed by the GSEAbase method ‘GeneSetCollection’ with the argument ‘setType = GOCollection()’. We used the GOstats method ‘HyperGTest’ to perform a hypergeometrical enrichment analysis for overrepresentation of GO terms in the set of significantly up- or downregulated genes as compared to all genes expressed in the condition and retrieved the results using the ‘summary’ method. The *p*-values were adjusted by the Benjamini-Hochberg multiple hypothesis testing correction with a significance level of 0.05.

### Cis-regulatory motif detection and constructing of gene regulatory networks

We searched for de novo cis-regulatory motifs using the RSAT peak-motifs tool in differential mode using default parameter settings [32]. Detected motifs were compared to the motif databases Cistrome (*A. thaliana* motifs detected by DAP-seq, 2016-06), footprintDB-plants (2020-01) and JASPAR core non-redundant plants (2020), in addition to cisBP (CIS-BP Database: Catalog of Inferred Sequence Binding Preferences) specific for *A. thaliana* (2015-06, v1.02) or *S. lycopersicum* (2019-06, v.2.00). We selected sequences of enriched TE family members near DE genes for the genomic positioning (upstream, downstream, within introns) for which an enrichment was found. All other TE sequences in the same genomic positioning were taken as background. In addition, we searched for known cis-regulatory motifs consisting of 5 bp at minimum, 2735 in total, collected from footprintDB [77], AGRIS, PLACE and the literature [54, 78], using the RSAT dna-pattern tool in all TE sequences in the different genomic regions for both species [31]. Cis-regulatory motif enrichment was calculated for each gene-proximal TE list with responsiveness to stress using hypergeometric enrichment against all TEs in the same genomic region with at least one motif and Benjamini-Hochberg multiple hypothesis testing correction with a confidence level of 95%. To further reduce the inclusion of false positives, we considered only motifs that were present in at least 10% of the TEs and that were at least two times enriched in the TE list compared to all TEs in that genomic positioning. For highly overrepresented motifs of known TFs, we visualized the TE-mediated networks for heat stress in Arabidopsis using Cytoscape 3.8.2.

## Supplementary Information

**Additional file 1.**

**Additional file 2.**

**Additional file 3.**

**Additional file 4.**

**Additional file 5.**

## Data Availability

The RNA-seq datasets analysed during the current study are available in the Sequence Read Archive (SRA) and BioProject repositories at NCBI, https://www.ncbi.nlm.nih.gov/bioproject/, with specific BioProject numbers available in Table S2 of the Supplementary Information. The genome datasets on transposable elements analysed in the current study are available at the The Arabidopsis Information Resource (TAIR) repository for *Arabidopsis thaliana*, https://www.arabidopsis.org/download/index-auto.jsp?dir=%2Fdownload_files%2FGenes%2FAraport11_genome_release%2Farchived, and at the Solanaceae Genomics Network repository for *Solanum lycopersicum*, ftp://ftp.solgenomics.net/tomato_genome/annotation/ITAG3.0_release/ .
