# Supplementary material for: Nearby transposable elements impact plant stress gene regulatory networks: a meta-analysis in A. thaliana and S. lycopersicum
Source: BMC Genomics. 2022 Jan 4;23:18. doi: 10.1186/s12864-021-08215-8 (PMC8725346; doi:10.1186/s12864-021-08215-8)
Supplement: Supplementary file 1 — Additional file 1. [file 12864_2021_8215_MOESM1_ESM.docx]

**Supplementary Information**

**Additional file 1**. Supplementary Data including:

- **Table S1.** Superfamilies of TEs (TEFs) adjacent to protein-coding genes in *A. thaliana* and *S. lycopersicum* and number of TEs present in these superfamilies.
- **Table S2.** Overview of the SRA data used in this study. For *A. thaliana* and *S. lycopersicum*, we started with respectively 20 and 33 experimental conditions. The strikethrough experimental conditions were filtered out upon preprocessing and filtering.
- **Figure S1.** Overview of the computational steps for preprocessing and differential expression analysis of the RNA-seq data. Top: The input was a RNA-seq reads file in fastq format, retrieved from the Sequence Read Archive (SRA). Tools: Trimmomatic 0.32, FastQC 0.11.2, GNSAP 2015-06-23, Samtools 1.3, HTSeq 0.6.1, Qualimap 2.1. Bottom: The input was a set of gene read counts corresponding to replicates of test and control conditions. Only genes exceeding a total number of counts across all input data larger than 10 plus the total number of datasets were included for analysis. Two tools detected differential expression in parallel, DESeq2 1.14.1 and EdgeR 3.16.5, from which the consensus was taken. If there were more than 100 differentially expressed (DE) genes with nearby transposons, further analysis was performed regarding the enrichment of TEs adjacent to DE genes.
- **Figure S2.** Comparison of distributions of log2 fold change (FC stress/control) values between all genes and genes located near certain TE families for Arabidopsis. The distribution of all genes is shown using a violin plot while the expression changes for individual genes near specific TEFs are shown using dots.

**Additional file 2.** **Table S3.** Overview of the TE superfamily (TEF)-differentially expressed genes enrichment analysis. For *A. thaliana* and *S. lycopersicum*, we list here the significant positive TEF-differentially expressed genes associations, the adjusted p-value by FDR, the enrichment score, the total number of expressed genes near the TE, the observed and expected frequencies of TE adjacent differentially expressed genes in the different stress conditions and genomic positionings. We additionally filtered out significant results for which the observed number of differentially expressed genes near a TEF was less than 5 and the expected number was less than 2. *See Excell table.*

**Additional file 3. Table S4.** Functional enrichment for the stress regulatory conditions with significant TE superfamily (TEF) associations. The 10 most significant GO Biological Process terms are shown, if available. *See Excell table.*

**Additional file 4. Table S5.** Overview of the known plant cis-regulatory motifs detected through RSAT dna-pattern and significantly overrepresented in the stress-responsive TE sequences of *A. thaliana* relative to all gene-proximal TE sequences in the same genomic positioning. We provided the percentage of TE sequences carrying the motif (perc), the motif enrichment score (enrich) and adjusted p-value (adj.p) (significance level 0.05) after hypergeometric enrichment with BH multiple hypothesis testing, as well as a motif name and description (desc). To further reduce the inclusion of false positives, we considered only motifs that were present in at least 10% of the TEs and that were at least two times enriched in the TE list compared to all TEs in that genomic positioning. *See Excell table.*

**Additional file 5. Table S6.** Overview of the known plant cis-regulatory motifs detected through RSAT dna-pattern and significantly overrepresented in the stress-responsive TE sequences of *S. lycopersicum* relative to all gene-proximal TE sequences in the same genomic positioning. We provided the percentage of TE sequences carrying the motif (perc), the motif enrichment score (enrich) and adjusted p-value (adj.p) (significance level 0.05) after hypergeometric enrichment with BH multiple hypothesis testing, as well as a motif name and description (desc). To further reduce the inclusion of false positives, we considered only motifs that were present in at least 10% of the TEs and that were at least two times enriched in the TE list compared to all TEs in that genomic positioning. *See Excell table.*

**Table S1.** Superfamilies of TEs (TEFs) adjacent to protein-coding genes in *A. thaliana* and *S. lycopersicum* and number of TEs present in these superfamilies.

| *A. thaliana* | |  | *S. lycopersicum* | |
| --- | --- | --- | --- | --- |
| Helitron | 7632 |  | Gypsy | 20936 |
| MuDR | 2729 |  | Copia | 14749 |
| Copia | 782 |  | LINE | 10701 |
| LINE | 740 |  | TIR_MITEov10 | 4527 |
| Gypsy | 605 |  | MuDR | 2641 |
| hAT | 602 |  | hAT | 2248 |
| SINE | 328 |  | CACTA | 969 |
| Pogo | 238 |  | EPRV | 729 |
| Harbinger | 201 |  | Harbinger | 703 |
| En-Spm | 187 |  | Mariner | 343 |
| ATREP19* | 135 |  | Retrotransposon | 309 |
| Mariner | 94 |  | Helitron | 284 |
| ATDNA12T3_2* | 68 |  | TRIM_LARD | 97 |
| Tc1 | 64 |  |  |  |
| ATREP18* | 15 |  |  |  |

*These are the most abundant TE families (>150 copies in the genome) for which the superfamily is currently ‘Unknown’ and which we considered as superfamilies for this study.

**Table S2.** Overview of the SRA data used in this study. For *A. thaliana* and *S. lycopersicum*, we started with respectively 20 and 33 experimental conditions. The strikethrough experimental conditions were filtered out upon preprocessing and filtering.

| **Bioproject** | **Species** | **Accession/Cultivar** | **#SRA** | **Experimental categories** | **Experimental conditions (#SRA for non-controls)** |
| --- | --- | --- | --- | --- | --- |
| PRJNA209324 | *A. thaliana* | Col-0 | 6 | drought | drought_A (4) |
| PRJNA217812 | *A. thaliana* | Col-0 | 31 | salt, salt_metal | ~~salt_A (6)~~, ~~salt_metal_A (6), salt_metal_B (6), salt_metal_C (5)~~ |
| PRJNA218632 | *A. thaliana* | Col-0 | 4 | cold | cold_A (2) |
| PRJNA284487 | *A. thaliana* | Col-0 | 6 | heat | ~~heat_A (4)~~ |
| PRJNA295091 | *A. thaliana* | Col-0 | 12 | salt, heat, salt_heat | salt_B (3), heat_B (3), salt_heat_A (3) |
| PRJNA309285 | *A. thaliana* | Col-0 | 24 | photorespiratory stress (mutants and/or stress) | photorespiratory_A (3), photorespiratory_mutant_A (3), photorespiratory_mutant_B (3), photorespiratory_mutant_C (3) |
| PRJNA309655 | *A. thaliana* | Col-0 | 6 | photorespiratory stress (mutants and/or stress) | ros_A (3) |
| PRJNA322265 | *A. thaliana* | Col-0 | 24 | proteasome stress (chemical inhibitors and mutants) | proteasome_inh_A (4), proteasome_ mutant_A (4), proteasome_mutant_B (4) |
| PRJNA336471 | *A. thaliana* | Col-0 | 48 | infection, paraquat | infection_A (12), paraquat_A (12) |
| PRJEB14805 | *S. lycopersicum* | Moneymaker | 6 | cold | cold_A (3) |
| PRJEB5335 | *S. lycopersicum* | Moneymaker | 4 | mycorrhizal symbiosis | ~~symbiosis_A (2)~~ |
| PRJNA291675 | *S. lycopersicum* | M82 | 96 | light | light_A (10), light_B (9), light_C (14), light_D (14) |
| PRJNA291401 | *S. lycopersicum* | CLN2777A | 6 | infection_viral (Tomato yellow leaf curl virus - TYLCV) | infection_viral_A (3) |
| PRJNA299687 | *S. lycopersicum* | Rio Grande prf3 | 20 | infection_effector (effector proteins *Pseudomonas syringae*) | infection_effector_A (4), infection_effector_B (4), infection_effector_C (4), infection_effector_D (4) |
| PRJNA319398 | *S. lycopersicum* | Moneymaker, Red Setter | 8 | heat | ~~heat_A (2)~~, heat_B (2) |
| PRJNA329199 | *S. lycopersicum* | RNAi–SlPH | 8 | infection_necrotrophic (*Colletotrichum gloeosporioides*) | infection_necrotrophic_A (4) |
| PRJNA339564 | *S. lycopersicum* | Rutgers | 4 | stress tolerant, male-sterile mutant | stress_tolerance_A (2) |
| PRJNA350553 | *S. lycopersicum* | Ailsa Craig | 4 | hormone (ethylene) | hormone_A (2) |
| PRJNA193536 | *S. lycopersicum* | Moneymaker | 6 | ~~infection_necrotrophic (~~*~~Botrytis cinerea~~*~~)~~ | ~~infection_necrotrophic_B (4)~~ |
| PRJNA214335 | *S. lycopersicum* | Rio Grande prf3 | 28 | infection_effector (flagellin), infection_necrotrophic (*Pseudomonas syringae* pv. tomato DC3000,  *Pseudomonas syringae* pv. DC3000 flagillin mutant,  *Pseudomonas syringae* pv. DC3000 AvrPto/AvrPtoB mutant), infection_nonpathogen (*Pseudomonas fluorescens*, *Pseudomonas putida*), ~~infection_biotrophic (~~*~~Agrobacterium tumefaciens~~*~~)~~ | infection_effector_E (3), infection_necrotrophic_C (3), infection_necrotrophic_mutant_A (3), infection_necrotrophic_mutant_B (3), infection_nonpathogen_A (3), infection_nonpathogen_B (3), ~~infection_biotrophic_A (3)~~ |
|  |  |  |  |  |  |
| PRJNA215996 | *S. lycopersicum* | Moneymaker, FL505 | 10 | ~~infection_viral (Tomato yellow leaf curl virus - TYLCV)~~ | ~~infection_viral_B (3), infection_viral_C (3)~~ |
| PRJNA217491 | *S. lycopersicum* | Micro-Tom | 6 | ~~infection_parasitic (~~*~~Rotylenchulus reniformis~~*~~)~~ | ~~infection_parasitic_A (3)~~ |
| PRJNA219445 | *S. lycopersicum* | Micro-Tom | 8 | hormone (ethylene, ethylene + auxin) | hormone_B (2), ~~hormone_C (2)~~ |
| PRJNA252525 | *S. lycopersicum* | Rio Grande | 18 | infection_necrotrophic | infection_necrotrophic_D (12) |
| PRJNA270083 | *S. lycopersicum* | Ailsa Craig | 12 | infection_necrotrophic, infection_necrotrophic_light (*Pseudomonas syringae* pv. tomato DC3000) | infection_necrotrophic_E (3), infection_necrotrophic_light_A (3) |
| PRJNA275562 | *S. lycopersicum* | Hazera 3042 | 10 | heat | heat_C (5) |


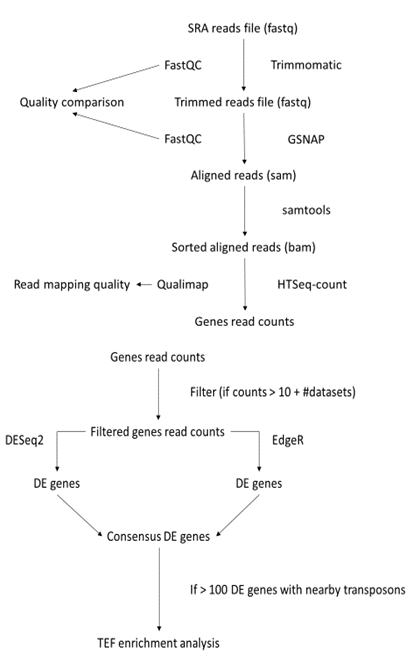


**Figure S1.** Overview of the computational steps for preprocessing and differential expression analysis of the RNA-seq data. Top: The input was a RNA-seq reads file in fastq format, retrieved from the Sequence Read Archive (SRA). Tools: Trimmomatic 0.32, FastQC 0.11.2, GNSAP 2015-06-23, Samtools 1.3, HTSeq 0.6.1, Qualimap 2.1. Bottom: The input was a set of gene read counts corresponding to replicates of test and control conditions. Only genes exceeding a total number of counts across all input data larger than 10 plus the total number of datasets were included for analysis. Two tools detected differential expression in parallel, DESeq2 1.14.1 and EdgeR 3.16.5, from which the consensus was taken. If there were more than 100 differentially expressed (DE) genes with nearby transposons, further analysis was performed regarding the enrichment of TEs adjacent to DE genes.


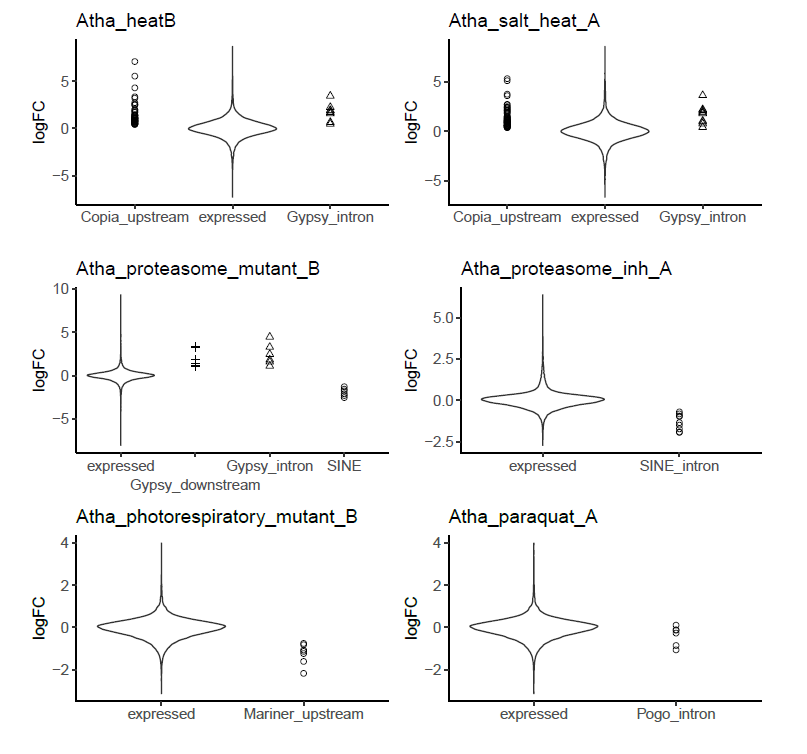


**Figure S2.** Comparison of distributions of log2 fold change (FC stress/control) values between all genes and genes located near certain TE families for Arabidopsis. The distribution of all genes is shown using a violin plot while the expression changes for individual genes near specific TEFs are shown using dots.
